# Pancreatic Schwannoma: A Rare Culprit But Accurate Diagnosis Can Avoid Resection

**DOI:** 10.7759/cureus.90115

**Published:** 2025-08-14

**Authors:** Shin Kato, Mariko Tsukamoto, Taichi Murai, Koji Hirata, Yuta Koike

**Affiliations:** 1 Department of Gastroenterology, Graduate School of Medicine, International University of Health and Welfare, Narita Hospital, Narita, JPN; 2 Department of Gastroenterology, Sapporo City General Hospital, Sapporo, JPN

**Keywords:** differential diagnosis of pancreatic tumors, endoscopic ultrasound-guided tissue acquisition, eus-fna, eus-ta, pancreatic schwannoma, s-100

## Abstract

Pancreatic schwannomas are extremely rare benign tumors originating from Schwann cells of peripheral nerves, often mimicking more common pancreatic tumors, such as neuroendocrine neoplasms or solid pseudopapillary neoplasms, making preoperative diagnosis challenging. We describe a 65-year-old asymptomatic man referred for evaluation of an incidental pancreatic body mass detected by ultrasound. Laboratory findings, including liver enzymes and tumor markers (CA19-9 and CEA), were normal. Contrast-enhanced CT revealed a well-defined 20 mm mass with delayed enhancement, and MRI showed low intensity on T1-weighted images, mildly high on T2, and slight diffusion restriction. Based on imaging, a pancreatic neuroendocrine neoplasm was initially suspected. Endoscopic ultrasound-guided tissue acquisition with a 22-gauge needle was performed. Histology revealed bundles of spindle-shaped cells with mild atypia and eosinophilic cytoplasm. Immunohistochemistry was diffusely positive for S-100 protein and negative for AE1/AE3, CD34, c-Kit, and desmin, confirming pancreatic schwannoma. Given its benign nature, the patient was managed conservatively without surgery, and no tumor growth was observed over two years of follow-up. This case highlights the importance of including schwannoma in the differential diagnosis of solid pancreatic lesions and illustrates how an endoscopic ultrasound (EUS)-guided biopsy with immunohistochemistry can enable accurate preoperative diagnosis and avoid unnecessary surgical resection.

## Introduction

Schwannomas are benign, slow-growing, encapsulated tumors derived from Schwann cells of the peripheral nerve sheaths and initially reported by Verocay in 1910 [1]. They typically arise in the head, neck, and mediastinum [2], while only 0.7% of schwannomas arise in the retroperitoneum, including the pancreas [3], with fewer than 100 cases reported in the English literature worldwide.

Radiologically, pancreatic schwannomas often present as well-defined solid or mixed solid-cystic lesions, mimicking pancreatic neuroendocrine neoplasm (PNEN), solid-pseudopapillary neoplasm (SPN), or even malignant tumors, such as adenocarcinoma or adenosquamous carcinoma [4,5]. Accurate preoperative diagnosis remains challenging but is clinically important, as most pancreatic schwannomas have a benign course and surgical resection can often be avoided.

## Case presentation

A 65-year-old asymptomatic man with no significant past medical history, family history of pancreatic cancer, or habits of smoking and drinking was referred to our hospital for further evaluation of a pancreatic body mass incidentally identified on a routine health check-up ultrasound.

**Figure 1:**
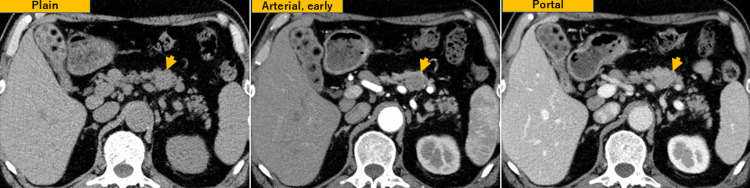
CT images Dynamic CT showed a 20 mm round, expansile mass (arrow head) with a smooth surface in the pancreatic body, without involvement of the main pancreatic duct. The mass was mildly enhanced in the early phase and showed gradual and marked enhancement in the delayed phase.

Laboratory tests showed no abnormalities, including liver function tests, serum amylase, lipase, and tumor markers (CA19-9 and CEA). Contrast-enhanced CT revealed a round, 20 mm, well-circumscribed mass in the pancreatic body with delayed enhancement and no involvement of the main pancreatic duct (Figure 1).

**Figure 2:**
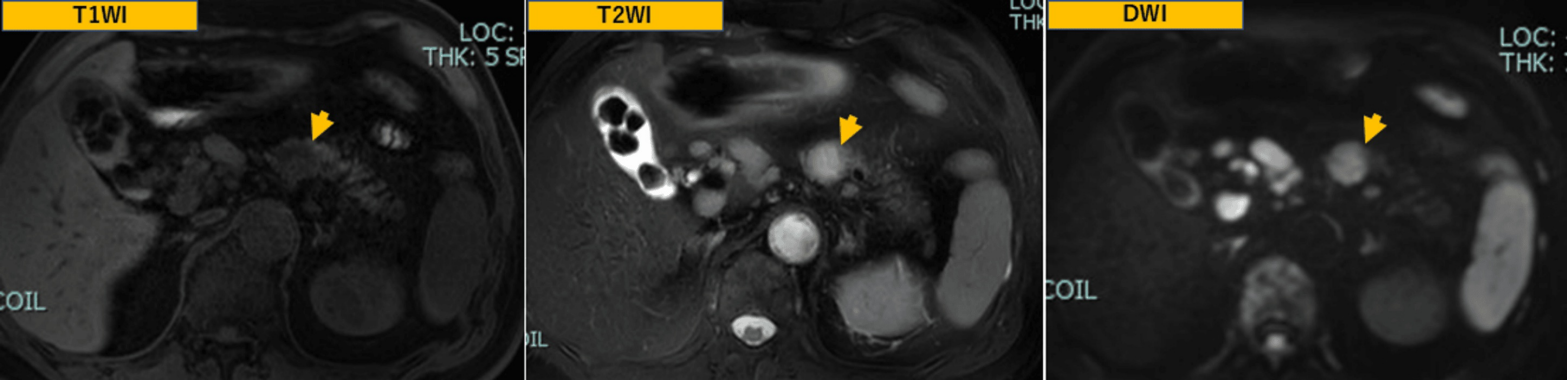
MRI images MRI demonstrated low intensity on T1-weighted images, mildly high intensity on T2-weighted images, and slight restriction on diffusion-weighted imaging of the mass (arrow head).

MRI demonstrated low intensity on T1-weighted images, mildly high intensity on T2-weighted images, and slight restriction on diffusion-weighted imaging of the mass (Figure 2).

Based on imaging, the differential diagnosis included PNEN (somewhat atypical because it was not strongly hypervascular), SPN, and adenosquamous carcinoma.

**Figure 3:**
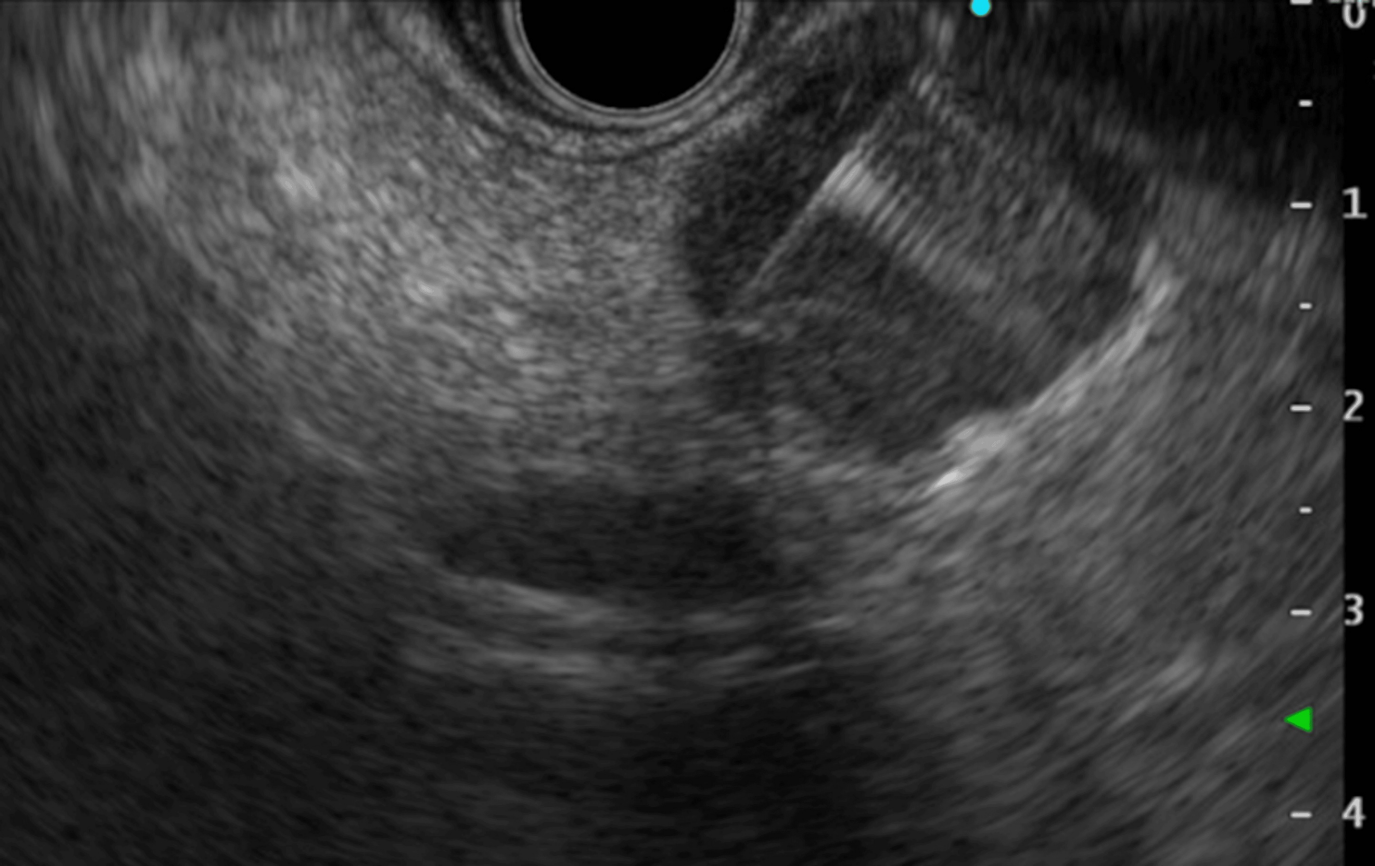
EUS image Endoscopic ultrasound (EUS) presented a low echoic, smooth surface mass on pancreatic body. EUS tissue acquisition (EUS-TA) was performed using a 22-gauge Franseen needle for two times puncture with aspiration.

For definitive diagnosis, endoscopic ultrasound-guided tissue acquisition (EUS-TA) was performed using a 22-gauge Franseen needle (SonoTip TopGain, Medico’s Hirata Inc., Osaka, Japan) (Figure 3).

**Figure 4:**
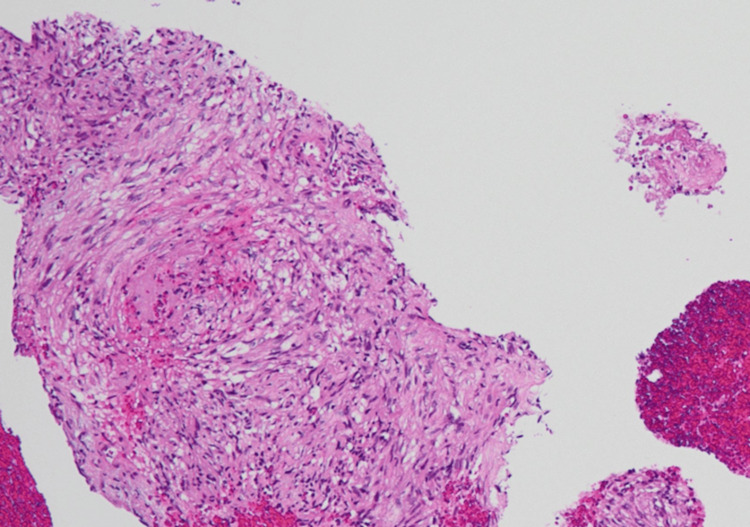
Hematoxylin-eosin stain Hematoxylin-eosin stain of specimen showed bundles of spindle-shaped cells with mildly atypical nuclei and clear to pale eosinophilic cytoplasm (magnification ×100).

Histopathological examination revealed bundles of spindle-shaped cells with mildly atypical nuclei and clear to pale eosinophilic cytoplasm (Figure 4).

**Figure 5:**
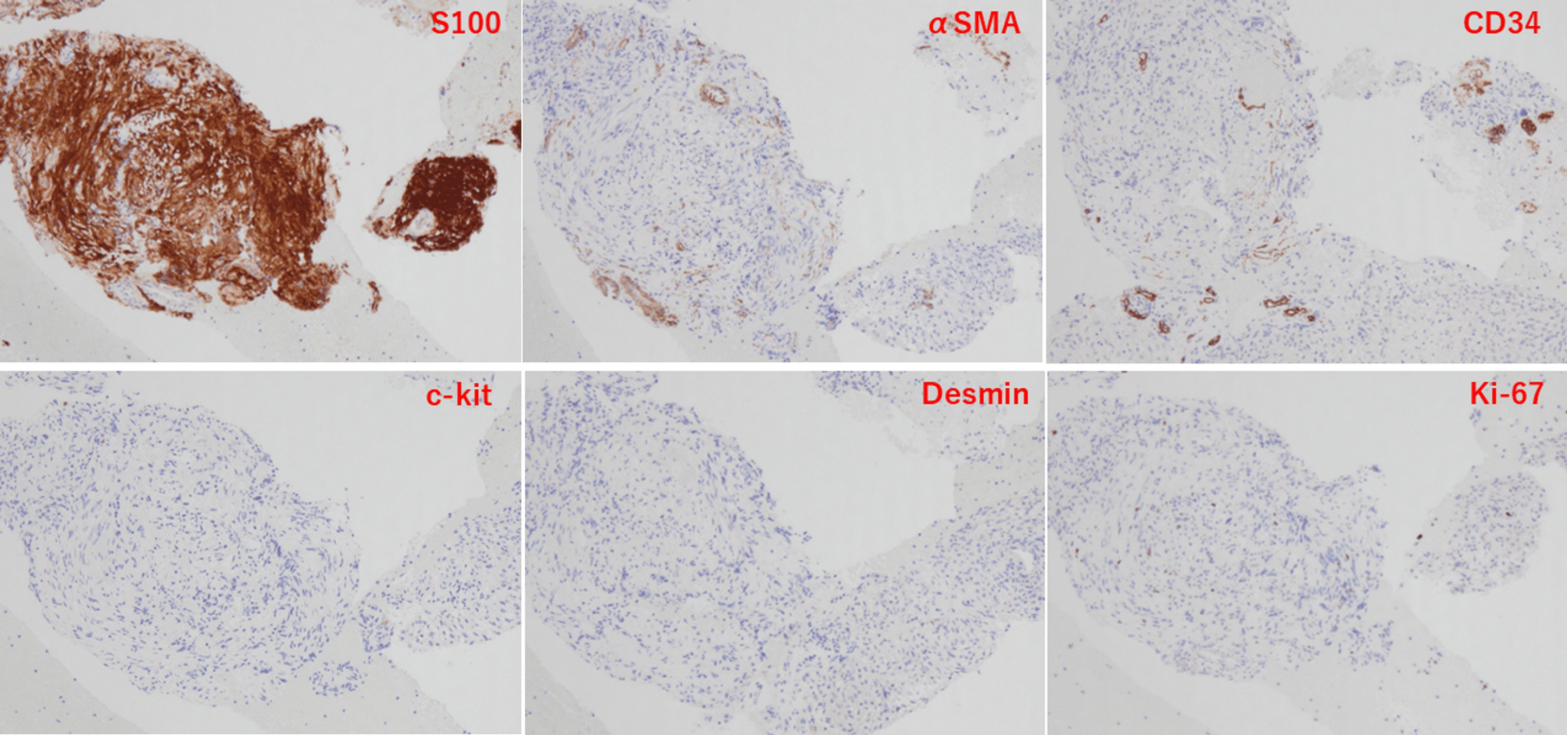
Immunohistochemical staining Immunohistochemical staining showed diffuse positivity for S-100 protein and negativity for αSMA, CD34, c-Kit, and desmin. Low expression of Ki-67 labelling index was also observed (magnification ×100).

Immunohistochemical staining showed diffuse positivity for S-100 protein and negativity for αSMA, CD34, c-Kit, Desmin, chromogranin A, and synaptophysin, consistent with schwannoma. Low expression of Ki-67 labelling index was also observed (Figure 5).

Given the benign nature of the pancreatic schwannoma and the absence of symptoms, the patient was managed conservatively with imaging surveillance instead of surgery. Over two years of follow-up, there was no evidence of tumor growth.

## Discussion

Pancreatic schwannomas are exceedingly rare, accounting for less than 1% of all pancreatic neoplasms [6,7]. These tumors often appear as round solid lesions, sometimes with necrosis or cystic degeneration [8], and may show a range of contrast enhancement patterns on CT, from hypervascular to delayed enhancement - all of which are nonspecific. On MRI, schwannomas present as homogeneously hypointensity on T1WI and hyperintense on T2WI. However, these findings sometimes appear heterogeneously hyperintense due to the variable degeneration background of the tumor, which is non-specific, similar to other pancreatic tumors [4,5]. Current report also described that pancreatic schwannomas frequently show increased FDG uptake on PET/CT, contrary to their benign features [9]. Because of their rarity and imaging overlap, they are frequently misdiagnosed as PNEN or SPN.

EUS-TA plays a critical role in the preoperative evaluation of pancreatic schwannomas [10-13]. The presence of spindle-shaped cells and a characteristic immunohistochemical profile - strong S-100 positivity and absence of epithelial and mesenchymal markers (AE1/AE3, c-Kit, CD34, and desmin) - helps confirm the diagnosis.

Accurate preoperative diagnosis is clinically important: while PNEN and SPN are often surgically resected due to malignant potential, pancreatic schwannomas are typically benign and slow-growing and rarely undergo malignant transformation [14]. There is no established index to strongly suspect malignancy unique character to pancreatic schwannoma, and the criteria for peripheral nerve and soft tissue-derived schwannomas have been used alternatively. According to these diagnostic criteria, findings suggestive of malignancy include tumor diameter greater than 5 cm, hemorrhagic necrosis, high Ki-67 labelling index, and a tendency to invade surrounding tissues [15]. Pancreatic schwannomas without these findings have a very high probability of being benign, and imaging follow-up rather than resection could be the gold standard. Thus, unnecessary surgery and its associated risks can be avoided through correct diagnosis.

Our case underscores the importance of including schwannomas in the differential diagnosis of solid pancreatic lesions and highlights the value of EUS-guided biopsy with immunohistochemistry for precise diagnosis.

## Conclusions

Pancreatic schwannomas, though rare, should be considered in the differential diagnosis of well-defined pancreatic masses. EUS-guided sampling with immunohistochemistry is essential for accurate preoperative diagnosis and may help avoid unnecessary surgical intervention.

## References

[1] Verocay J (1910). [On the knowledge of "neurofibromas"]. Beitr Z Path Anat.

[2] Schultz E, Sapan MR, McHeffey-Atkinson B, Naidich JB, Arlen M (1994). Case report 872. "Ancient" schwannoma (degenerated neurilemoma). Skeletal Radiol.

[3] Das Gupta TK, Brasfield RD, Strong EW, Hajdu SI (1969). Benign solitary schwannomas (neurilemomas). Cancer.

[4] Gupta A, Subhas G, Mittal VK, Jacobs MJ (2009). Pancreatic schwannoma: literature review. J Surg Educ.

[5] Aichouni N, Abbou W, Nasri S (2021). Pancreatic schwannoma- CT and MRI findings: a rare case report and review of literature. Ann Med Surg (Lond).

[6] Almo KM, Traverso LW (2001). Pancreatic schwannoma: an uncommon but important entity. J Gastrointest Surg.

[7] Ercan M, Aziret M, Bal A (2016). Pancreatic schwannoma: a rare case and a brief literature review. Int J Surg Case Rep.

[8] Park JS, Min SJ, Kim H, Choi JA (2021). Pancreatic schwannoma with cystic degeneration: a case report and literature review. Taehan Yongsang Uihakhoe Chi.

[9] Fukuhara S, Fukuda S, Tazawa H (2017). A case of pancreatic schwannoma showing increased FDG uptake on PET/CT. Int J Surg Case Rep.

[10] Hanaoka T, Okuwaki K, Imaizumi H (2021). Pancreatic schwannoma diagnosed by endoscopic ultrasound-guided fine-needle aspiration. Intern Med.

[11] Azami T, Takano Y, Niiya F (2020). A case of primary pancreatic schwannoma diagnosed by endoscopic ultrasound-fine needle aspiration. Clin J Gastroenterol.

[12] Doxtader EE, Sturgis CD, Dyhdalo KS (2018). Cystic pancreatic schwannoma diagnosed by endoscopic ultrasound-guided fine needle aspiration. Diagn Cytopathol.

[13] Sung S, Rao R, Sharaiha RZ, Halazun KJ, Elsoukkary S, Hoda RS (2017). Fine-needle aspiration cytology of pancreatic schwannoma. Diagn Cytopathol.

[14] Ma Y, Shen B, Jia Y (2017). Pancreatic schwannoma: a case report and an updated 40-year review of the literature yielding 68 cases. BMC Cancer.

[15] Woodruff JM, Kourea HP, Louis DN, Scheithauer BW (2000). Pathology and Genetics of Tumours of the Nervous System. WHO Classification of Tumours, 3rd Edition, Volume 1. Pathology and Genetics: Tumours of the Nervous System.

